# Lipotoxicity: Effects of Dietary Saturated and Transfatty Acids

**DOI:** 10.1155/2013/137579

**Published:** 2013-01-31

**Authors:** Débora Estadella, Claudia M. da Penha Oller do Nascimento, Lila M. Oyama, Eliane B. Ribeiro, Ana R. Dâmaso, Aline de Piano

**Affiliations:** ^1^Programa de Pós-Graduação em Nutrição, Disciplina de Fisiologia da Nutrição, EPM, Universidade Federal de São Paulo (UNIFESP), Rua Botucatu, 862 Edifício de Ciências Biomédicas, 2 andar, Vila Clementino, 04023-060 São Paulo, SP, Brazil; ^2^Departamento de Biociências, UNIFESP, Campus Baixada Santista, 11060-001 Santos, SP, Brazil

## Abstract

The ingestion of excessive amounts of saturated fatty acids (SFAs) and transfatty acids (TFAs) is considered to be a risk factor for cardiovascular diseases, insulin resistance, dyslipidemia, and obesity. The focus of this paper was to elucidate the influence of dietary SFA and TFA intake on the promotion of lipotoxicity to the liver and cardiovascular, endothelial, and gut microbiota systems, as well as on insulin resistance and endoplasmic reticulum stress. The saturated and transfatty acids favor a proinflammatory state leading to insulin resistance. These fatty acids can be involved in several inflammatory pathways, contributing to disease progression in chronic inflammation, autoimmunity, allergy, cancer, atherosclerosis, hypertension, and heart hypertrophy as well as other metabolic and degenerative diseases. As a consequence, lipotoxicity may occur in several target organs by direct effects, represented by inflammation pathways, and through indirect effects, including an important alteration in the gut microbiota associated with endotoxemia. Interactions between these pathways may perpetuate a feedback process that exacerbates an inflammatory state. The importance of lifestyle modification, including an improved diet, is recommended as a strategy for treatment of these diseases.

## 1. Introduction

Fat is an important component of the normal human diet. It is a source of energy and provides essential fatty acids and fat-soluble vitamins. However, several fatty acids in fats, especially saturated fatty acids (SFAs) and trans fatty acids (TFAs) may have adverse effects on human health [1–3]. 

In the human diet, SFAs are derived from animal sources, while TFAs originate in meat and milk from ruminant animals and result from bacterial biohydrogenation of unsaturated fatty acids in the rumen [4]. In addition, partial hydrogenation of unsaturated fatty acids in vegetable oils during the industrial production of certain foods produces TFA [5]. Small amounts of TFA are produced during the processes used to deodorize or refine vegetable oils to make the products more stable and robust and thus easier to handle or store [6, 7].

Most TFAs have physical properties similar to SFAs [8]. More specifically, monounsaturated TFA isomers with 18-carbon chain lengths (trans-18 : 1) are among the most predominant TFAs present in the human diet [9, 10]. It is well established that intake of SFA and TFA is a significant risk factor for cardiovascular diseases (CVD) as well as inflammation, insulin resistance, and obesity. These fatty acids also induce endothelial dysfunction and an unfavorable blood lipid profile, including increased LDL-c and decreased HDL-c levels [2, 11, 12].

High SFA and TFA intake, the typical dietary pattern of western populations, favors a proinflammatory status that contributes to development of insulin resistance. Roles for SFA and TFA intake have been demonstrated in several inflammatory pathways and result from imbalances in the highly interconnected lipid signaling pathways that contribute to disease progression in chronic inflammation, autoimmunity, allergy, cancer, atherosclerosis, hypertension, and heart hypertrophy as well as metabolic and degenerative diseases [13, 14]. 

The focus of this paper was to elucidate the influence and role of dietary SFA and TFA intake in lipotoxicity in the liver and the cardiovascular, endothelial, and gut microbiota systems as well as in insulin resistance and endoplasmatic reticulum stress.

## 2. Insulin Sensitivity and Resistance

Insulin is an anabolic hormone that exerts several important metabolic effects. Insulin regulates glucose homeostasis at several levels, including decreasing hepatic glucose synthesis and increasing peripheral glucose uptake, primarily in muscle and adipose tissue. Moreover, this hormone stimulates lipogenesis and the synthesis of protein in hepatic and adipose tissues, while reducing lipolysis and proteolysis [15].

Events that occur after insulin binds to its receptor are highly regulated and specific and can be influenced by numerous factors such as the dietary composition, including the quantity and type of fatty acids [16, 17]. 

Although several mechanisms have been implicated in the development of insulin resistance [16], more studies are necessary to elucidate the link between the mechanisms of insulin resistance and fatty acid intake.

Increased lipid availability reduces insulin-stimulated glucose consumption in skeletal muscle. This effect is generally explained as a fatty acid-mediated inhibition of insulin signaling [15]. Moreover, in a recent investigation it was shown that a palatable hyperlipidic diet, rich with SFA, causes obesity and affects brain glucose metabolism in rats [18].

In a clinical study, short-term elevation of free fatty acids (FFAs) induced insulin resistance, which occurs primarily at the cellular level in skeletal muscle [17].

A chronic increase in plasma FFA levels is harmful as shown by the important effects of these dietary components in pancreatic beta cell lipotoxicity. Fatty acid derivatives can interfere with the function of these cells and ultimately lead to their death through lipoapoptosis [19].

Fatty acids in excess not only induce hepatic insulin resistance but also impair insulin clearance *in vitro* and *in vivo* in animals [20, 21] and humans [22]. This leads to the typical hyperinsulinemia observed in insulin-resistant states and in nonalcoholic fatty liver disease (NAFLD) [23, 24].

Several studies performed by our group have demonstrated that long-term interdisciplinary therapy reduces fat intake, in particular SFA, in obese adolescents. The intervention resulted in decreased visceral fat and tumor necrosis factor-alpha (TNF-*α*) and interleukin-6 (IL-6) levels and increased levels of interleukin-10 (IL-10) and adiponectin, accompanied by a reduction in homeostatic model assessment-insulin resistance (HOMA-IR) and the occurrence of associated diseases [25–28]. In the context of these results, we proposed that the altered insulinemic status could be considered to play a key role in the development of several cardiometabolic risk comorbidities [12, 29–31]. 

In one review article, fatty acids were said to affect insulin secretion as a function of the chain length. Thus, insulin secretion increases as a function of longer carbon chains and decreases as a function of the degree of unsaturation. These findings suggest that SFA and TFA influence insulin resistance [32].

In an animal model, although TFA ingestion had no effect on fasting plasma glucose, insulin levels, or oral glucose tolerance, it significantly decreased insulin-stimulated glucose uptake in muscles compared to polyunsaturated fatty acids (PUFAs) [33].

In a cross-sectional study of individuals with high cardiometabolic risk, the association of TFA intake with insulin resistance was demonstrated. The authors speculated that TFA interferes with insulin signaling predominantly via intracellular kinases, which alter insulin receptor substrates [34]. 

Activation of intracellular kinases, such as inhibitor of nuclear factor-*κ*B kinase (IKK) and c-Jun N-terminal kinase (JNK), alters insulin receptor substrates and decreases insulin sensitivity. Additionally, it is important to note that activation of transcription factors can contribute to reduced glucose uptake by the expression of proinflammatory cytokines, such as TNF-alpha and IL-6, causing impairment in the insulin receptor phosphorylation (Figure 3) [35].

The deleterious role of SFA in glucose and lipid metabolism has been previously shown. A partial explanation is that SFA increases production of cytokines such as TNF-*α* and IL-6 through hypertrophic adipocytes and infiltrating macrophages, and these cytokines cause the deterioration of insulin sensitivity [35].

Treatment of primary mouse hepatocytes and pancreatic cells with palmitic acid, an SFA, caused sustained JNK activation and insulin resistance. Moreover, the palmitic acid treatment inhibited glucose-induced insulin gene transcription. This effect may be mediated by interference of autocrine insulin signaling through the phosphorylation of insulin receptor substrates 1 and 2 at sites that interfere with their binding to activated insulin receptors [36]. It has been proposed that long-chain saturated fatty acids, such as palmitic acid, can trigger insulin resistance in both primary hepatocytes and pancreatic *β*-cells in a JNK-dependent manner. The JNK phosphorylation site on IRS-2 may be functionally equivalent to Ser-307 of IRS-1 (Figure 3). Moreover, JNK might also be involved in negative regulation of insulin synthesis or signaling within the pancreatic *β*-cells, the central site of blood glucose regulation. Thus, palmitic acid can be considered to be a potent activator of JNK in cultured hepatocytes and *β*-cells, leading to IRS-1 and IRS-2 Ser/Thr phosphorylation [36].

## 3. Hepatic Injury

The emergence of obesity has led to substantial prevalence of NAFLD. Worldwide, the prevalence of NAFLD ranges between 10% and 39%. However, the prevalence of NAFLD is increased in certain populations: the condition affects approximately 50% of diabetics, 57% to 74% of the obese, and up to 90% of morbidly obese people. Therefore, in the last decade, this disease has been recognized as an emerging clinical problem in obese patients [37, 38]. The pathogenesis of NAFLD is unclear, but there is evidence that insulin resistance, inflammation, and genetic and dietary factors, as well as lifestyle, exert key roles in the development of NAFLD [39].

**Figure 1:**
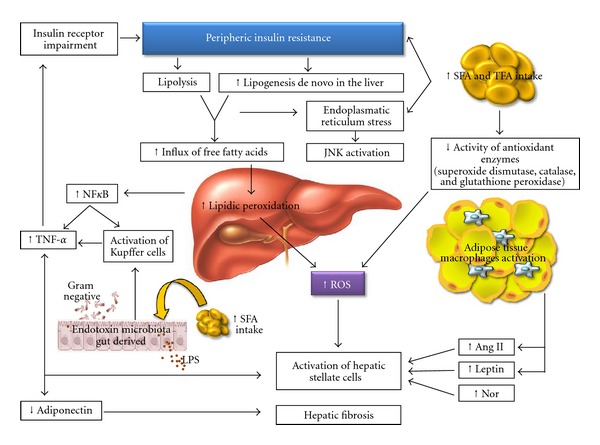
Schematic representation of SFA and TFA excess intake effects in hepatic injury and in endotoxemia. Ang II: angiotensin II, JNK: Jun N-terminal kinase, LPS: lipopolysaccharides, NF-*κ*B: nuclear factor kappa-B, Nor: Noradrenaline, ROS: reactive oxygen species, SFA: saturated fatty acids, TFA: trans fatty acids, and TNF-*α*: tumor necrosis factor-alpha.

Several studies have revealed an association between obesity and NAFLD progression. In NAFLD patients, the adipocytes of the visceral tissue demonstrate elevated lipolytic activity, promoting a high influx of FFA into the portal vein [37, 38, 40]. Moreover, the literature shows an association between visceral adipocytes and the hepatic cellular inflammatory process [41]. It is believed that the physiopathology of NAFLD may be driven by several forms of hepatic injury. The first hypothesis involved in the development of NAFLD is related to insulin resistance. The influence of genetic and environmental factors can promote peripheral insulin resistance, which leads to increased levels of nonesterified fatty acids (NEFAs). Subsequently, a high influx of these fatty acids into hepatocytes promotes increased hepatic de novo lipogenesis. When the rate of lipogenesis exceeds the rate of *β*-oxidation of fatty acids and the exportation of VLDL, hepatic fat accumulation is observed (Figure 1) [40].

The exact mechanisms that promote the progression from steatosis to steatohepatitis have not yet been completely elucidated. However, it is clear that apoptosis can be a pathophysiologic marker of nonalcoholic steatohepatitis in some steatotic patients. 

It has been proposed that the accumulation of FFA, especially saturated fatty acids, in the hepatocytes can promote apoptosis by diverse pathways. These may include ROS-induced stress that affects the mitochondrial membranes, endoplasmic reticulum, and lysosomes. Lipid peroxidation increases the levels of reactive oxygen species, which may be partially responsible for hepatocyte dysfunction [42] (Figure 1).

Apoptosis of the hepatocytes can occur via extrinsic or intrinsic pathways. The extrinsic pathway is induced by death ligands such as Fas (a key death receptor belonging to the tumor-necrosis-factor- (TNF-) receptor family) and TRAIL (TNF-related apoptosis-inducing ligand). In contrast, the intrinsic pathway of cell death is activated by the intracellular stress of membrane-bound organelles, such as ER, lysosomes, and mitochondria [43].

Saturated fatty acids activate complex intracellular pathways, including the activation of Toll-like receptor 4 (TLR-4), which subsequently stimulates TNF-*α* production. TNF-*α* consequently activates the JNK pathway [40], leading to the upregulation of the proapoptotic BH3-only protein PUMA (p53-upregulated modulator of apoptosis). PUMA then associates with BIM (Bcl-2-interacting mediator of cell death) to activate BAX, a proapoptotic protein of the BCL-2 (B-cell lymphoma 2) family. The end result of this pathway is mitochondrial dysfunction, activation of the caspase cascade, and cell death [43].

The TFA obtained in the diet accumulates in cellular triacylglycerols and phospholipids. Chronically excessive triacylglycerol accumulation in tissues such as the liver, muscle, and pancreatic beta cells leads to a protective response involving adaptation of the adipocytes, and this response includes activation of several inflammatory pathways that promote adipose tissue insulin resistance [34]. 

Toll-like receptors (TLRs) are classical pattern recognition receptors of the innate immune response. Two of these receptors, TLR4 and TLR2, are activated by SFA but inhibited by docosahexaenoic acid [44].

TLR4 activates both MyD88-dependent and -independent pathways. The MyD88-dependent pathway is initiated by the recruitment of TIRAP, an adaptor between the TIR domain of TLR-4 and MyD88, followed by activation of the IRAK family of protein kinases and subsequent phosphorylation of TRAF6. In the MyD88-independent pathway, the activation of TLR-4 requires the participation of TRAM to recruit TRIF, which then activates TRAF6 and TRAF3. TRAF6, directly or via TAK1, stimulates the IKK complex which promotes phosphorylation of IkB leading to activation of nuclear factor *κ*B (NF-*κ*B) (Figure 3) [45]. 

FFAs activate TLR-4 receptors in macrophages and adipocytes, which results in increased proinflammatory cytokine gene and protein expression [46]. Recently, it has been demonstrated that saturated fatty acids can induce TNF-*α* expression by macrophages by activating the MyD88-independent pathway [47]. 

TLR4 activation plays a central role in the inflammatory process, activating inflammatory cytokine gene transcription and also inducing endoplasmic reticulum stress. The activation of TLR4 by LPS induces a potent endoplasmic reticulum stress and unfolded protein response through the PERK/eIF2*α* and IRE1*α*/XBP1 pathways (Figure 4) [48]. NFkB, a transcription factor known as a mediator of immune and antiapoptotic responses, is activated by the accumulation of membrane proteins in the ER. Specifically, the accumulated proteins lead to the production of reactive oxygen intermediates, which activate NFkB by degradation of IkB. Recently, it has been proposed that the phosphorylation of eIF2a is required for the triggering of NFkB. In summary, the apoptotic process involves the activation of the gene for C/EBP homologous protein (CHOP), also known as growth arrest and DNA damage-inducible gene 153 (GADD153) (Figure 4); the activation of the cJUN NH2-terminal kinase (JNK) pathway, which is mediated by the formation of the inositol-requiring 1 (Ire1)-TNF receptor-associated factor 2 (TRAF2)-apoptosis signal-regulating kinase1 (ASK1) complex; and the activation of the ER-associated caspase-12. In humans, these apoptotic pathways eventually lead to the activation of caspase-3 [49]. 

The second hypothesis of NAFLD physiopathology refers to the release of fatty acids from dysfunctional and insulin-resistant adipocytes. This release causes lipotoxicity, mainly to visceral adipose tissue [40]. The visceral adipose tissue is infiltrated by inflammatory cells, including macrophages and other immune cells, which increase secretion of inflammatory adipokines such as leptin, IL-6, TNF-*α*, and angiotensinogen, as well as reducing secretion of adiponectin, an anti-inflammatory adipokine [50]. Insulin resistance of the visceral adipose tissue is associated with increased lipolytic activity, which increases the levels of FFA in the portal circulation, potentially resulting in hepatotoxicity (Figure 1) [40, 41]. 

Experimentally, it has been demonstrated that the decreased secretion of adiponectin in obesity alters lipid metabolism and insulin sensitivity in the liver. However, administration of recombinant adiponectin to adiponectin-deficient obese mice fed a high-fat diet dramatically alleviated hepatomegaly, steatosis, and inflammation [51]. 

At the clinical level, adipose tissue insulin resistance contributes to type 2 diabetes mellitus and CVD. On the other hand, decreasing plasma FFA concentration by administration of acipimox, a nicotinic acid analogue that inhibits adipose tissue lipolysis, rapidly improves muscle insulin sensitivity [52].

The potential ability of FFA to alter skeletal muscle glucose metabolism was first proposed more than 50 years ago [53] and has been widely investigated. The FFA-induced effects on this tissue do not appear to be the result of the accumulation of intramyocellular lipids per se. Rather, skeletal muscle insulin resistance is closely correlated with the presence of a variety of toxic metabolites derived from incomplete oxidation of fatty acids, such as acylcarnitines and long-chain fatty acyl CoAs, ceramides, and/or diacylglycerols [54, 55].

Oxidative stress is believed to be an important factor in the development of NAFLD [56]. The importance of fatty acids in this process is clear from the observation that biological membranes adjust their composition according to the fatty acid content of dietary fat [57]. Dietary fatty acids can influence the susceptibility of cells to oxidative stress, perhaps due to changes in the fatty acid composition of the cellular membranes [58].

Intake of high levels of SFA is associated with increased lipid content in the liver [18, 59–61] and with liver dysfunction. This dysfunction is thought to be caused by an increase in the production in reactive oxygen species, which leads to damage of the hepatic mitochondria. In addition, SFA intake exceeding 10% of total energy promotes insulin resistance, which plays a key role in the development of NAFLD [62].

The association between high intake of SFA and cholesterol with NAFLD has been previously demonstrated [63, 64]. SFAs promote endoplasmic reticulum stress as well as hepatocyte injury. Accumulation of SFA in the liver leads to an increase in markers associated with endoplasmic reticulum stress, such as reactive oxygen species and caspase activation. These biomarkers are associated with liver dysfunction. Moreover, the positive correlation between SFA intake and insulin resistance, which plays a key role in the development of NAFLD, has been demonstrated. These correlations suggest that limiting SFA intake is a valuable nutritional strategy for the prevention and treatment of NAFLD [62]. 

Papandreou et al. [64] demonstrated that the SFA intake was directly proportional to the degree of hepatic steatosis. In their study, multiple regression analysis of factors associated with fatty liver showed that HOMA-IR and SFA were the most significant factors for this condition after adjustment for age, gender, and diet. We have also observed an association among SFA intake, NAFLD, and orexigenic neuropeptides [26]. 

Diets rich in fatty acids, mainly SFA and TFA, as well as carbohydrate-rich diets, favor an acute increase in insulin resistance independent of adiposity. High SFA intake may also promote steatohepatitis directly by modulating hepatic triacylglycerol accumulation and oxidative activity and indirectly by affecting insulin sensitivity and postprandial triacylglycerol metabolism [64]. 

In clinical studies performed by our research group, we showed a positive correlation between calories derived from SFA intake and visceral fat in NAFLD patients [23, 26]. These data suggested that composition of the diet exerts an important role in the development of NAFLD and its treatment and that it is essential to consider excessive SFA intake as a critical risk factor for development of NAFLD [26].

Another potentially important mechanism for lipotoxicity of TFA in the liver is their effects on hepatic antioxidant enzymes. Reactive oxygen species (ROS) form as natural byproducts from the normal metabolism of oxygen and have important roles in cell signaling and homeostasis. However, during times of environmental stress, ROS levels can increase dramatically. This may result in significant damage to cell structures. Cumulatively, this is known as oxidative stress. Endogenous antioxidant enzymes, including superoxide dismutase (SOD), catalase (CAT), and glutathione peroxidase (GPx), are essential to defend against ROS. The removal of reactive oxygen substances is accomplished by enzymatic and nonenzymatic reactions in biological systems. In enzymatic reactions, SOD converts superoxide anions to hydrogen peroxide (H_2_O_2_), and H_2_O_2_ can be rapidly degraded by CAT and GPx to H_2_O [65–67].

High-fat diets can cause the formation of toxic intermediates that can inhibit the activity of antioxidant enzymes, resulting in the accumulation of O_2_ radicals and H_2_O_2_, which subsequently form hydroxyl radicals [68, 69]. TFAs are associated with a decrease in the efficiency of the antioxidant-enzymatic system and therefore with the increase of oxidative stress in rat livers. TFA may impart their effect by enhancing intrinsic signaling mechanisms leading to a chronic, pro-inflammatory state. Consumption of diets high in TFA may induce long-term progressive changes in the antioxidant enzyme's activities [70].

**Figure 2:**
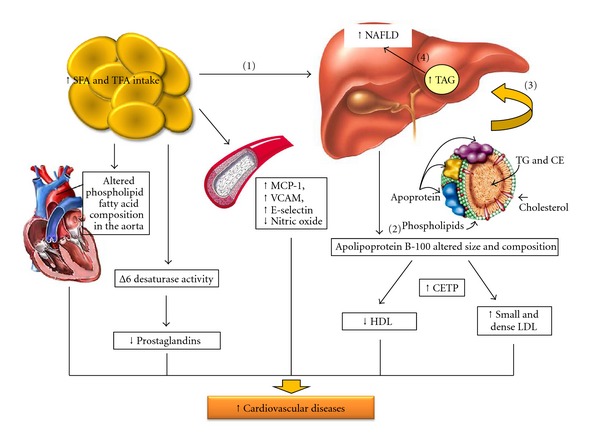
Schematic representation of SFA and TFA excess intake effects in hepatic injury, lipid metabolism, and cardiovascular risk. CE: cholesterol esterified, CETP: cholesteryl ester transfer protein, HDL: high density cholesterol, LDL: low density cholesterol, MCP-1: monocyte chemotactic protein-1, NAFLD: nonalcoholic fatty liver disease, SFA: saturated fatty acids, TFA: trans fatty acids, TG: triacylglycerol, and VCAM: vascular cell adhesion molecule.

**Figure 3:**
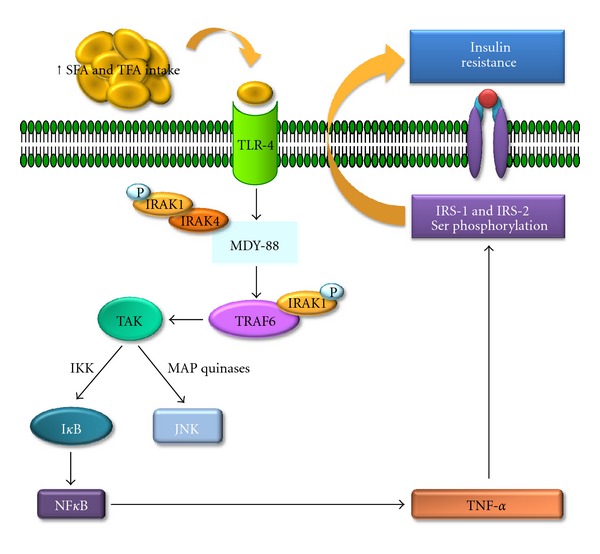
Schematic representation of SFA and TFA excess intake effects in the mechanisms of insulin resistance development. IKB: inhibitor of nuclear factor-*κ*B, IKK: inhibitor of nuclear factor-*κ*B kinase, IRAK-1: interleukin-1 receptor-associated kinase 1, IRAK-4: interleukin-1 receptor-associated kinase 4, IRS-1: insulin receptor substrate-1, IRS-2: insulin receptor substrate-2, JNK: Jun N-terminal kinase, MAP kinases: mitogen activated protein kinases, MDY-88: myeloid differentiation primary response gene (88), NF-*κ*B: nuclear factor kappa B, P: phosphorus, TAK: thylakoid arabidopsis kinase, TLR-4: Toll-like receptor-4, TNF-*α*: tumor necrosis factor-alpha, TRAF-6: receptor-associated factor 6.

Finally, another pathway by which excessive TFA intake could cause hepatic injury is through effects on lipid metabolism. *In vitro*, TFAs alter the secretion, lipid composition, and size of apolipoprotein B-100 (apoB-100) particles produced by hepatic cells [71] (Figure 2). Specifically, these cells fail to synthesize the apolipoprotein, which is required to package and export fat from the liver. Therefore, the liver accumulates triacylglycerol. In a study in which male Wistar rats were fed a high-fat diet including 20% fresh soybean oil diet, 20% oxidized soybean oil diet, or 20% margarine diet for 4 weeks, the highest inflammatory response in the liver was induced by the margarine diet. The authors demonstrated that oxidized edible oils fed to rats for four weeks increased lipid peroxidation in the liver compared with rats fed nonoxidized oils. These results suggest that a strong relationship exists between the consumption of TFA in the oxidized oils and lipid peroxidation. This study provides evidence for a direct effect of TFA on liver dysfunction caused by disturbances in hepatic lipid metabolism. The resulting NAFLD is a key component of cardiometabolic risk. This evidence suggests that TFA may influence the risk factors for CVD [70].

Thus, the link between dysfunctional adipocytes and the liver involves several pathways that combine to promote the development of lipotoxic liver disease, a term that more accurately describes the pathophysiology of nonalcoholic steatohepatitis. 

In fact, hepatic steatosis is considered to be a hepatic manifestation of cardiometabolic risk. This condition is associated with obesity, insulin resistance, hypertension, and dyslipidemia. Clinical studies corroborate the relationship between NAFLD and CVD. Specifically, obese NAFLD patients were shown to have a greater intima-media thickness (IMT), a subclinical marker of the atherosclerotic process, compared to non-NAFLD patients [72]. In this context, our research group performed a study that involved one year of interdisciplinary intervention in obese adolescents. The results indicated that the improvement of HOMA-IR was an independent predictor of carotid IMT changes in this population [30]. As previously discussed, hyperinsulinemia from increased insulin secretion and decreased insulin clearance correlates with the severity of hepatic steatosis, and chronically elevated plasma insulin levels may promote atherogenesis [73, 74]. Hyperglycemia, *per se*, and the typical atherogenic dyslipidemia in NAFLD driven by oversecretion of VLDL are established factors for CVD. However, whether NAFLD and CVD are mechanistically related or merely both associated with lipotoxicity remains to be established. 

## 4. Cardiovascular Risk

Studies suggest multiple possible mechanisms that might mediate the association of TFA with CVD. Three main pathways for these physiological effects have been proposed: serum lipid concentrations, systemic inflammation, and endothelial cell function [3, 75].

Consumption of industrial TFA increases the blood concentrations of low-density lipoprotein (LDL), triacylglycerols, and Lp(a) lipoprotein while decreasing the levels of high-density lipoprotein (HDL) and reducing the particle size of LDL cholesterol. Furthermore, consumption of TFA can increase the ratio of total cholesterol to HDL cholesterol, a powerful predictor of CVD risk [3, 76]. Thus, TFAs have markedly adverse effects on serum lipid profiles.

As described previously, there is an important relationship between intake of TFA and incidence of CVD. However, the effects exceed those predicted by the changes in serum lipids alone, suggesting that TFAs influence other risk factors for CVD [77]. Specifically, in addition to their effects on lipid/lipoprotein profiles, TFA consumption is known to influence multiple risk factors including increased systemic inflammation [78], increased thrombogenesis, and reduced endothelial function [2], all of which, in combination or individually, contribute to increased cardiovascular risk. Experimental studies suggest that TFAs exert their multiple effects by influencing metabolic and signaling pathways in hepatocytes, monocytes, adipocytes, and endothelial cells. The precise molecular pathways through which TFAs influence these cell types are not well established [77].

The effect of TFA on systemic inflammation can be partially explained by the influence of these fatty acids on the prostaglandin balance. The effects on these processes can influence thrombogenesis and impair the activity of Δ desaturase, the enzyme responsible for the conversion of linoleic acid to arachidonic acid and other n-6 PUFA. Thus, this inhibition alters essential fatty acid metabolism [79]. Moreover, in an animal model of excess TFA consumption, changes in the phospholipid fatty acid composition in the aorta were observed [80] (Figure 2). TFAs have been associated with the activation of systemic inflammatory responses, including substantially increased levels of IL-6, plasminogen activator inhibitor-1 (PAI-1), TNF-*α*, TNF receptors, and monocyte chemoattractant protein-1, and with increased levels of several markers of endothelial activation, such as soluble intercellular adhesion molecule 1, soluble vascular-cell adhesion molecule 1, and E-selectin [2, 3, 81] (Figure 2). 

In controlled trials, however, TFA did not increase all inflammatory markers [78, 82]. Oxidative stress may also explain the high risk of CVD associated with industrial TFA intake [83].

Oxidative stress induced by free radicals has been associated with the development of several diseases including CVDs, most likely through a vascular proinflammatory response [84]. However, further research is necessary to fully elucidate the implications of the effects of TFA on some markers of oxidative stress. Although the possible mechanisms that link TFA and oxidative stress are unknown, efforts to eliminate partially hydrogenated oils from the diet remain necessary and important to reduce the burden of CVD [70]. 

The third pathway linking TFA and CVD refers to the possible influence of TFA on endothelial cell function. Endothelial nitric oxide synthase (eNOS) synthesizes nitric oxide (NO) in response to many stimuli, such as fluid shear stress and insulin. These stimuli increase NO production in endothelial cells through an insulin receptor substrate-1 (IRS-1-) and phosphatidylinositol 3-kinase (PI3-kinase-) dependent pathway that causes phosphorylation of endothelial nitric oxide synthase (eNOS) by Akt [85]. 

A review of the influences of fatty acids on endothelial cell function suggested that increased ingestion of fatty acids impairs endothelial cell insulin signaling and NO production through the activation of the IKK/NF-*κ*B pathway. Furthermore, an experimentally induced elevation of the concentration of plasma FFA in humans alters endothelial function [46].

It has also been shown that the dietary SFA palmitate attenuates endothelial insulin signaling and NO production by first activating NF-*κ*B signaling, which results in a reduction in IRS-1/pAkt/peNOS signaling [77]. 

The reduction of SFA intake is considered a primary goal for decreasing the risk of CVD. A low SFA diet was demonstrated to be associated with the reduced progression of coronary atherosclerosis [86, 87]. 

The effect of SFA intake on the plasma lipid risk factors and effects on CVD are similar to those described for TFA intake. However, SFA ingestion is particularly associated with activation of the TLR pathways. 

In a clinical investigation, TLR-4 and TLR-2 expression and activity were increased in the monocytes of patients with cardiometabolic risk. The pathways regulated by these receptors could contribute to the patients' high risk for CVD [88].

## 5. Endoplasmic Reticulum Stress

Lipid peroxidation is defined by a biochemical cascade that results in oxidative degradation of PUFA. When the lipid peroxidation occurs in biological membranes, it causes impaired membrane function and structural integrity, decreases in membrane fluidity, and inactivation of several membrane enzymes [89]. Niu et al. [90] reported that phospholipids derived from TFA had a higher membrane cholesterol affinity than their *cis*-analogues. Thus, TFA ingestion could alter cell membrane structure, organization, and composition in an ROS-mediated manner.

A recent animal experiment indicated that TFA reduced the membrane fluidity of fat cells and impaired cell function. The suggested mechanism involved production of additional reactive oxygen species associated with the increase in lipid peroxidation in the groups fed the TFA diet [91]. A high-fat diet induces endoplasmic reticulum stress (ER), which activates IKK and JNK, thereby impairing insulin signaling [92]. 

Recent evidence suggests that lipotoxicity in hepatocytes involves ER stress and JNK-mediated apoptosis [93, 94].

**Figure 4:**
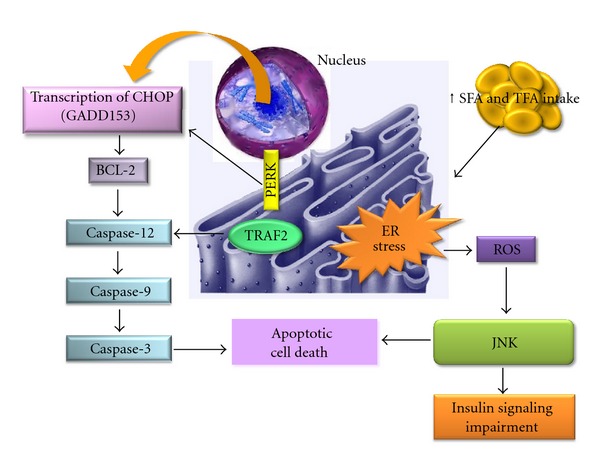
Schematic representation of SFA and TFA excess intake effects in endoplasmatic reticulum stress. BCL-2: B-cell lymphoma 2, CHOP: CCAAT/-enhancer-binding protein homologous protein, ER: endoplasmatic reticulum, GAAD 153: DNA damage-inducible gene 153, JNK: Jun N-terminal kinase, PERK: protein kinase RNA-like endoplasmic reticulum kinase, ROS: reactive oxygen species, SFA: saturated fatty acids, TFA: trans fatty acids, and TRAF2: TNF receptor-associated factor 2.

Disturbances in the normal functions of the ER lead to an evolutionarily conserved cell stress response, the unfolded protein response, which is aimed initially at compensating for damage but can eventually trigger cell death if ER dysfunction is severe or prolonged. Although the mechanisms by which ER stress leads to cell death are not completely understood, some of them have been described in the literature. A study of mice deficient in caspase-12 showed that while the cells of these mice were resistant to ER stress-induced apoptosis, apoptosis of the cells occurred normally in response to other death stimuli [95]. Based on these data, it was proposed that other pathways leading to cell death by ER stress should be explored. Increases in apoptotic proteins, such as BIM, BAK, and PUMA, were observed during ER stress, suggesting a connection between stress signals and the proapoptotic switch that occurs when cellular homeostasis is irreversibly altered, finally leading to cell death [96, 97]. The ER responds to the burden of unfolded proteins in its lumen by activating intracellular signal transduction pathways, generically termed the unfolded protein response (UPR). Another suggested mechanism is that the three UPR branches provide opposing signals and that the relative timing of their induction shifts the balance between cytoprotection and apoptosis as unmitigated ER stress persists. Specifically, IRE1 signaling attenuates upon prolonged ER stress, and PERK (protein kinase RNA-like endoplasmic reticulum kinase) signaling induces its own deactivation via GADD34 expression (Figure 4). Both pathways thus contain intrinsic timers that are likely to contribute to the life-or-death decision [98]. 

Important roles for ER-initiated cell death pathways have been recognized for several diseases, including hypoxia, ischemia/reperfusion injury, neurodegeneration, heart disease, and diabetes [99].

Studies suggest that cytokines, as well as elevated lipids, especially long-chain SFA, may induce ER stress in pancreatic *β*-cells and liver cells. SFA-induced *β*-cell death has been shown to be related to the activation of caspases [94, 100]. Elevated lipids also induce apoptosis in a number of cell types, suggesting that ER stress may be an early component of lipotoxicity [94].

A study of cultured H4IIE liver cells investigated the influence of SFA and TFA in the apoptosis process and the role of the ER stress-induced activation of caspases. The authors observed that SFA induced ER stress and increased both caspase-9 and caspase-3 activity (Figure 4). The authors hypothesized that saturation, *per se*, plays a role in lipotoxicity in liver cells [94].

## 6. Gut Microbiota

The human gut contains a massive number of microorganisms or microbiota. Several mechanisms have been proposed to link gut flora to obesity, including the role of the gut microbiota in increasing energy extraction from indigestible dietary polysaccharides [101] and elevating plasma lipopolysaccharide levels, resulting in chronic low-grade inflammation [102].

The intestinal flora exerts an important role in normal gut function and maintenance of health, and the dietary composition can influence the sequence and the nature of colonization. Cani et al. [103] found that a high-fat diet resulted in a significant change in the composition of the dominant bacterial populations within the gut microflora, including a decrease in the number of *Bifidobacteria*, Eubacterium, rectal *Clostridium coccoides* group, and *Bacteroides*, thus favoring an increase in the gram-negative to gram-positive ratio. This change in gut microflora composition was associated with a significant increase in plasma lipopolysaccharide (LPS) levels, fat mass, body weight gain, liver hepatic triglyceride accumulation, insulin resistance, and diabetes [103, 104]. In addition, de Wit et al. [105] observed that a high saturated fatty acid diet enhanced an overflow of dietary fat to the distal intestine, which affected the gut microbiota composition. This alteration was associated with obesity development and hepatic steatosis. 

The gut microbiota of obese individuals or those consuming a high content of saturated fatty acids contains predominantly gram-negative bacteria rich in LPS. Toll-like receptors in the cell membranes recognize LPS in the circulation (endotoxemia) and activate specific kinases, which lead to insulin resistance. These pathways also activate NF-*κ*B, which results in the expression of inflammatory genes. Similar to LPS, saturated fatty acids are also recognized by membrane receptors that trigger proinflammatory signaling pathways [102, 106]. 

Recently, our group demonstrated a positive correlation between plasma endotoxin concentration and both proinflammatory cytokines (especially IL-6) and insulin resistance in obese adolescents. Importantly, after long-term (one year) interdisciplinary therapy, endotoxemia, proinflammatory status, and insulin resistance were decreased [25]. These results showed the effectiveness of making lifestyle changes (i.e., nutritional modification) in reducing the proinflammatory state in obese individuals [107, 108].

## 7. Conclusion

**Figure 5:**
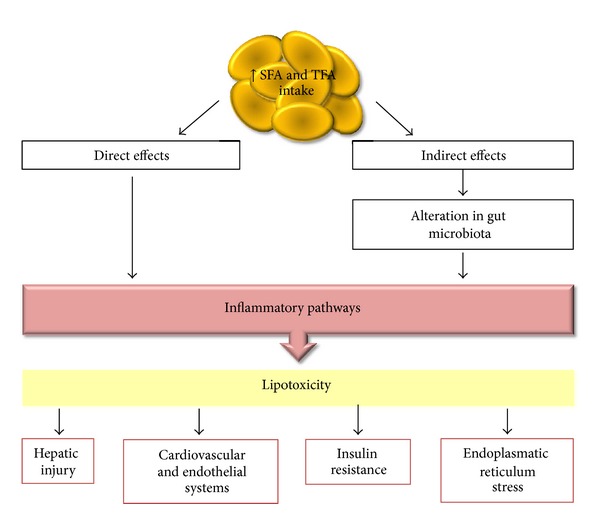
Schematic representation of SFA and TFA excess intake effects in the development of lipotoxicity in several target organs.

These experimental and clinical findings indicate that excess intake of both SFA and TFA can promote lipotoxicity in several target organs by direct effects, represented by inflammatory pathways, and indirect effects, including important alterations in the gut microbiota with implications for the endotoxemia process (Figure 5). Interplay between these pathways perpetuates a feedback process in which an inflammatory state elevates the risk factors for diverse diseases.

## References

[1] Mensink RP, Zock PL, Kester ADM, Katan MB (2003). Effects of dietary fatty acids and carbohydrates on the ratio of serum total to HDL cholesterol and on serum lipids and apolipoproteins: a meta-analysis of 60 controlled trials. *The American Journal of Clinical Nutrition*.

[2] Lopez-Garcia E, Schulze MB, Meigs JB (2005). Consumption of trans fatty acids is related to plasma biomarkers of inflammation and endothelial dysfunction. *Journal of Nutrition*.

[3] Mozaffarian D, Katan MB, Ascherio A, Stampfer MJ, Willett WC (2006). Trans fatty acids and cardiovascular disease. *The New England Journal of Medicine*.

[4] Bauman DE, Mather IH, Wall RJ, Lock AL (2006). Major advances associated with the biosynthesis of milk. *Journal of Dairy Science*.

[5] Ledoux M, Juanéda P, Sébédio J-L (2007). Trans fatty acids: definition and occurrence in foods. *European Journal of Lipid Science and Technology*.

[6] Tasan M, Demirci M (2003). Trans FA in sunflower oil at different steps of refining. *Journal of the American Oil Chemists’ Society*.

[7] Obara N, Fukushima K, Ueno Y (2010). Possible involvement and the mechanisms of excess trans-fatty acid consumption in severe NAFLD in mice. *Journal of Hepatology*.

[8] Ohlrogge JB, Emken EA, Gulley RM (1981). Human tissue lipids: occurrence of fatty acid isomers from dietary hydrogenated oils. *Journal of Lipid Research*.

[9] Eckel RH, Borra S, Lichtenstein AH, Yin-Piazza SY, Trans Fat Conference Planning Group (2007). Understanding the complexity of trans fatty acid reduction in the American diet: American heart association trans fat conference 2006: report of the trans fat conference planning group. *Circulation*.

[10] van de Vijver LP, Kardinaal AF, Couet C (2000). Association between trans fatty acid intake and cardiovascular risk factors in Europe: the TRANSFAIR study. *European Journal of Clinical Nutrition*.

[11] Sun Q, Ma J, Campos H (2007). A prospective study of trans fatty acids in erythrocytes and risk of coronary heart disease. *Circulation*.

[12] Dâmaso AR, de Piano A, Sanches PL (2011). Hyperleptinemia in obese adolescents deregulates neuropeptides during weight loss. *Peptides*.

[13] Wymann MP, Schneiter R (2008). Lipid signalling in disease. *Nature Reviews Molecular Cell Biology*.

[14] do Nascimento CMO, Ribeiro EB, Oyama LM (2009). Metabolism and secretory function of white adipose tissue: effect of dietary fat. *Anais da Academia Brasileira de Ciencias*.

[15] Rorsman P, Braun M (2012). Regulation of insulin secretion in human pancreatic islets. *Annual Review of Physiology*.

[16] Carvalheira JBC, Zecchin HG, Saad MJA (2002). Vias de Sinalização da Insulina. *Arquivos Brasileiros De Endocrinologia and Metabologia*.

[17] Szendroedi J, Frossard M, Klein N (2012). Lipid-induced insulin resistance is not mediated by impaired transcapillary transport of insulin and glucose in humans. *Diabetes*.

[18] Estadella D, Oyama LM, Bueno AA (2011). A palatable hyperlipidic diet causes obesity and affects brain glucose metabolism in rats. *Lipids in Health and Disease*.

[19] Unger RH, Clark GO, Scherer PE, Orci L (2010). Lipid homeostasis, lipotoxicity and the metabolic syndrome. *Biochimica et Biophysica Acta*.

[20] Svedberg J, Strömblad G, Wirth A, Smith U, Björntorp P (1991). Fatty acids in the portal vein of the rat regulate hepatic insulin clearance. *Journal of Clinical Investigation*.

[21] Wiesenthal SR, Sandhu H, McCall RH (1999). Free fatty acids impair hepatic insulin extraction in vivo. *Diabetes*.

[22] Carpentier A, Mittelman SD, Bergman RN, Giacca A, Lewis GF (2000). Prolonged elevation of plasma free fatty acids impairs pancreatic *β*-cell function in obese nondiabetic humans but not in individuals with type 2 diabetes. *Diabetes*.

[23] de Piano A, Prado WL, Caranti DA (2007). Metabolic and nutritional profile of obese adolescents with nonalcoholic fatty liver disease. *Journal of Pediatric Gastroenterology and Nutrition*.

[24] Tock L, Dâmaso AR, de Piano A (2010). Long-term effects of metformin and lifestyle modification on nonalcoholic Fatty liver disease obese adolescents. *Journal of Obesity*.

[25] Lira FS, Rosa JC, Pimentel GD (2012). Long-term interdisciplinary therapy reduces endotoxin level and insulin resistance in obese adolescents. *Nutrition Journal*.

[26] de Piano A, Tock L, Carnier J (2010). The role of nutritional profile in the orexigenic neuropeptide secretion in nonalcoholic fatty liver disease obese adolescents. *European Journal of Gastroenterology and Hepatology*.

[27] de Piano A, de Mello MT, Sanches LP (2012). Long-term effects of aerobic plus resistance training on the adipokines and neuropeptides in nonalcoholic fatty liver disease obese adolescents. *European Journal of Gastroenterology and Hepatology*.

[28] Campos RM, de Piano A, da Silva PL (2012). The role of pro/anti-inflammatory adipokines on boné metabolism in NAFLD obese adolescents: effects of long-term interdisciplinary therapy. *Endocrine*.

[29] Caranti DA, Tock L, Prado WL (2007). Long-term multidisciplinary therapy decreases predictors and prevalence of metabolic syndrome in obese adolescents. *Nutrition, Metabolism and Cardiovascular Diseases*.

[30] de Lima Sanches P, de Mello MT, Elias N (2011). Improvement in HOMA-IR is an independent predictor of reduced carotid intima-media thickness in obese adolescents participating in an interdisciplinary weight-loss program. *Hypertension Research*.

[31] Corgosinho FC, de Piano A, Sanches PL (2012). The role of PAI-1 and adiponectin on the inflammatory state and energy balance in obese adolescents with metabolic syndrome. *Inflammation*.

[32] Haber EP, Curi R, Carvalho CRO, Carpinelli AR (2001). Secreção da insulina: efeito autócrino da insulina e modulação por ácidos graxos. *Arquivos Brasileiros De Endocrinologia and Metabologia*.

[33] Jeyakumar SM, Prashant A, Rani KS (2011). Chronic consumption of trans-fat-rich diet increases hepatic cholesterol levels and impairs muscle insulin sensitivity without leading to hepatic steatosis and hypertriglyceridemia in female fischer rats. *Annals of Nutrition and Metabolism*.

[34] Angelieri CT, Barros CR, Siqueira-Catania A, Ferreira SRG (2012). Trans fatty acid intake is associated with insulin sensitivity but independently of inflammation. *Brazilian Journal of Medical and Biological Research*.

[35] Kennedy A, Martinez K, Chuang CC, Lapoint K, Mcintosh M (2009). Saturated fatty acid-mediated inflammation and insulin resistance in adipose tissue: mechanisms of action and implications. *Journal of Nutrition*.

[36] Solinas G, Naugler W, Galimi F, Lee MS, Karin M (2006). Saturated fatty acids inhibit induction of insulin gene transcription by JNK-mediated phosphorylation of insulin-receptor substrates. *Proceedings of the National Academy of Sciences of the United States of America*.

[37] Angulo P (2002). Treatment of nonalcoholic fatty liver disease. *Annals of Hepatology*.

[38] Festi D, Colecchia A, Sacco T, Bondi M, Roda E, Marchesini G (2004). Hepatic steatosis in obese patients: clinical aspects and prognostic significance. *Obesity Reviews*.

[39] Zivkovic AM, German JB, Sanyal AJ (2007). Comparative review of diets for the metabolic syndrome: implications for nonalcoholic fatty liver disease. *The American Journal of Clinical Nutrition*.

[40] Lam B, Younossi ZM (2010). Review: treatment options for nonalcoholic fatty liver disease. *Therapeutic Advances in Gastroenterology*.

[41] Ayonrinde OT, Olynyk JK, Beilin LJ (2011). Gender-specific differences in adipose distribution and adipocytokines influence adolescent nonalcoholic fatty liver disease. *Hepatology*.

[42] Jaeschke H (2011). Reactive oxygen and mechanisms of inflammatory liver injury: present concepts. *Journal of Gastroenterology and Hepatology*.

[43] Ibrahim SH, Kohli R, Gores GJ (2011). Mechanisms of lipotoxicity in NAFLD and clinical implications. *Journal of Pediatric Gastroenterology and Nutrition*.

[44] Huang S, Rutkowsky JM, Snodgrass RG (2012). Saturated fatty acids activate TLR-mediated proinflammatory signaling pathways. *Journal of Lipid Research*.

[45] Mohan C, Zhu J (2010). Toll-like receptor signaling pathways—Therapeutic opportunities. *Mediators of Inflammation*.

[46] Cusi K (2012). Role of obesity and lipotoxicity in the development of nonalcoholic steatohepatitis: pathophysiology and clinical implications. *Gastroenterology*.

[47] Youssef-Elabd EM, McGee KC, Tripathi G (2012). Acute and chronic saturated fatty acid treatment as a key instigator of the TLR-mediated inflammatory response in human adipose tissue, in vitro. *Journal of Nutritional Biochemistry*.

[48] Denis RG, Arruda AP, Romanatto T (2010). TNF-*α* transiently induces endoplasmic reticulum stress and an incomplete unfolded protein response in the hypothalamus. *Neuroscience*.

[49] Oyadomari S, Mori M (2004). Roles of CHOP/GADD153 in endoplasmic reticulum stress. *Cell Death and Differentiation*.

[50] Chitturi S, Wong VW, Farrell G (2011). Nonalcoholic fatty liver in Asia: firmly entrenched and rapidly gaining ground. *Journal of Gastroenterology and Hepatology*.

[51] Xu A, Wang Y, Keshaw H, Xu LY, Lam KSL, Cooper GJS (2003). The fat-derived hormone adiponectin alleviates alcoholic and nonalcoholic fatty liver diseases in mice. *Journal of Clinical Investigation*.

[52] Cusi K, Kashyap S, Gastaldelli A, Bajaj M, Cersosimo E (2007). Effects on insulin secretion and insulin action of a 48-h reduction of plasma free fatty acids with acipimox in nondiabetic subjects genetically predisposed to type 2 diabetes. *The American Journal of Physiology, Endocrinology and Metabolism*.

[53] Randle PJ, Garland PB, Hales CN, Newsholme EA (1963). The glucose fatty-acid cycle: its role in insulin sensitivity and the metabolic disturbances of diabetes mellitus. *The Lancet*.

[54] Holland WL, Brozinick JT, Wang LP (2007). Inhibition of ceramide synthesis ameliorates glucocorticoid-, saturated-fat-, and obesity-induced insulin resistance. *Cell Metabolism*.

[55] Watson ML, Coghlan M, Hundal HS (2009). Modulating serine palmitoyl transferase (SPT)expression and activity unveils a crucial role in lipid-induced insulin resistance in rat skeletal muscle cells. *Biochemical Journal*.

[56] Videla LA, Rodrigo R, Araya J, Poniachik J (2006). Insulin resistance and oxidative stress interdependency in non-alcoholic fatty liver disease. *Trends in Molecular Medicine*.

[57] Quiles JL, Huertas JR, Battino M (2002). The intake of fried virgin olive or sunflower oils differentially induces oxidative stress in rat liver microsomes. *British Journal of Nutrition*.

[58] Nakbi A, Tayeb W, Grissa A (2010). Effects of olive oil and its fractions on oxidative stress and the liver's fatty acid composition in 2,4-Dichlorophenoxyacetic acid-treated rats. *Nutrition and Metabolism*.

[59] Estadella D, Oyama LM, Dâmaso AR, Ribeiro EB, Oller Do Nascimento CM (2004). Effect of palatable hyperlipidic diet on lipid metabolism of sedentary and exercised rats. *Nutrition*.

[60] Starling RD, Trappe TA, Parcell AC, Kerr CG, Fink WJ, Costill DL (1997). Effects of diet on muscle triglyceride and endurance performance. *Journal of Applied Physiology*.

[61] Taguchi H, Omachi T, Nagao T, Matsuo N, Tokimitsu I, Itakura H (2002). Dietary diacylglycerol suppresses high fat diet-induced hepatic fat accumulation and microsomal triacylglycerol transfer protein activity in rats. *Journal of Nutritional Biochemistry*.

[62] Cave M, Deaciuc I, Mendez C (2007). Nonalcoholic fatty liver disease: predisposing factors and the role of nutrition. *Journal of Nutritional Biochemistry*.

[63] Allard JP, Aghdassi E, Mohammed S (2008). Nutritional assessment and hepatic fatty acid composition in non-alcoholic fatty liver disease (NAFLD): a cross-sectional study. *Journal of Hepatology*.

[64] Papandreou D, Rousso I, Malindretos P (2008). Are saturated fatty acids and insulin resistance associated with fatty liver in obese children?. *Clinical Nutrition*.

[65] Das UN (2010). A defect in Δ6 and Δ5 desaturases may be a factor in the initiation and progression of insulin resistance, the metabolic syndrome and ischemic heart disease in South Asians. *Lipids in Health and Disease*.

[66] Das UN (2011). A defect in the activities of Δ and Δ desaturases and pro-resolution bioactive lipids in the pathobiology of non-alcoholic fatty liver disease. *World Journal of Diabetes*.

[67] Das UN (2013). Nonalcoholic fatty liver disease as a pro-resolution defective disorder. *Nutrition*.

[68] Thampi BS, Manoj G, Leelamma S, Menon VP (1991). Dietary fiber and lipid peroxidation: effect of dietary fiber on levels of lipids and lipid peroxides in high fat diet. *Indian Journal of Experimental Biology*.

[69] Batra S, Singh SP, Srivastava VM, Chatterjee RK (1989). Xanthine oxidase, superoxide dismutase, catalase and lipid peroxidation in Mastomys natalensis: effect of Dipetalonema viteae infection. *Indian Journal of Experimental Biology*.

[70] Dhibi M, Brahmi F, Mnari A (2011). The intake of high fat diet with different trans fatty acid levels differentially induces oxidative stress and non alcoholic fatty liver disease (NAFLD) in rats. *Nutrition and Metabolism*.

[71] Mitmesser SH, Carr TP (2005). Trans fatty acids alter the lipid composition and size of apoB-100-containing lipoproteins secreted by HepG2 cells. *Journal of Nutritional Biochemistry*.

[72] Gökçe S, Atbinici Z, Aycan Z, Cinar HG, Zorlu P The relationship between pediatric nonalcoholic fatty liver disease and cardiovascular risk factors and increased risk of atherosclerosis in obese children.

[73] Ortiz-Lopez C, Lomonaco R, Orsak B (2012). Prevalence of prediabetes and diabetes and metabolic profile of patients with nonalcoholic fatty liver disease (NAFLD). *Diabetes Care*.

[74] Lorenzo C, Hanley AJ, Wagenknecht LE (2013). Relationship of insulin sensitivity, insulin secretion, and adiposity with insulin clearance in a multiethnic population: the insulin resistance atherosclerosis study. *Diabetes Care*.

[75] Ascherio A (2002). Epidemiologic studies on dietary fats and coronary heart disease. *The American Journal of Medicine*.

[76] Wanders AJ, Brouwer IA, Siebelink E, Katan MB (2010). Effect of a high intake of conjugated linoleic acid on lipoprotein levels in healthy human subjects. *PLoS ONE*.

[77] Iwata NG, Pham M, Rizzo NO, Cheng AM, Maloney E, Kim F (2011). Trans fatty acids induce vascular inflammation and reduce vascular nitric oxide production in endothelial cells. *PLoS ONE*.

[78] Baer DJ, Judd JT, Clevidence BA, Tracy RP (2004). Dietary fatty acids affect plasma markers of inflammation in healthy men fed controlled diets: a randomized crossover study. *The American Journal of Clinical Nutrition*.

[79] Kinsella JE, Bruckner G, Mai J, Shimp J (1981). Metabolism of trans fatty acids with emphasis on the effects of trans,trans-octadecadienoate on lipid composition, essential fatty acid, and prostaglandins: an overview. *The American Journal of Clinical Nutrition*.

[80] Kummerow FA, Zhou Q, Mahfouz MM, Smiricky MR, Grieshop CM, Schaeffer DJ (2004). Trans fatty acids in hydrogenated fat inhibited the synthesis of the polyunsaturated fatty acids in the phospholipid of arterial cells. *Life Sciences*.

[81] Pisani LP, Oller do Nascimento CM, Bueno AA (2008). Hydrogenated fat diet intake during pregnancy and lactation modifies the PAI-1 gene expression in white adipose tissue of offspring in adult life. *Lipids in Health and Disease*.

[82] Lichtenstein AH, Erkkilä AT, Lamarche B, Schwab US, Jalbert SM, Ausman LM (2003). Influence of hydrogenated fat and butter on CVD risk factors: remnant-like particles, glucose and insulin, blood pressure and C-reactive protein. *Atherosclerosis*.

[83] Smit LA, Katan MB, Wanders AJ, Basu S, Brouwer IA (2011). A high intake of trans fatty acids has little effect on markers of inflammation and oxidative stress in humans. *Journal of Nutrition*.

[84] Harvey KA, Arnold T, Rasool T, Antalis C, Miller SJ, Siddiqui RA (2008). Trans-fatty acids induce pro-inflammatory responses and endothelial cell dysfunction. *British Journal of Nutrition*.

[85] Montagnani M, Ravichandran LV, Chen H, Esposito DL, Quon MJ (2002). Insulin receptor substrate-1 and phosphoinositide-dependent kinase-1 are required for insulin-stimulated production of nitric oxide in endothelial cells. *Molecular Endocrinology*.

[86] Griel AE, Kris-Etherton PM (2006). Beyond saturated fat: the importance of the dietary fatty acid profile on cardiovascular disease. *Nutrition Reviews*.

[87] Sacks FM, Katan M (2002). Randomized clinical trials on the effects of dietary fat and carbohydrate on plasma lipoproteins and cardiovascular disease. *The American Journal of Medicine*.

[88] Jialal I, Huet BA, Kaur H, Chien A, Devaraj S (2012). Increased toll-like receptor activity in patients with metabolic syndrome. *Diabetes Care*.

[89] Lima ÉS, Abdalla DSP (2001). Peroxidação lipídica: mecanismos e avaliação em amostras biológicas. *Brazilian Journal of Pharmaceutical Sciences*.

[90] Niu SL, Mitchell DC, Litman BJ (2005). Trans fatty acid derived phospholipids show increased membrane cholesterol and reduced receptor activation as compared to their cis analogs. *Biochemistry*.

[91] Ibrahim A, Natarajan S, Ghafoorunissa (2005). Dietary trans-fatty acids alter adipocyte plasma membrane fatty acid composition and insulin sensitivity in rats. *Metabolism*.

[92] Tobar N, Oliveira AG, Guadagnini D (2011). Diacerhein improves glucose tolerance and insulin sensitivity in mice on a high-fat diet. *Endocrinology*.

[93] Zhang Y, Yang X, Shi H, Dong L, Bai J (2011). Effect of *α*-linolenic acid on endoplasmic reticulum stress-mediated apoptosis of palmitic acid lipotoxicity in primary rat hepatocytes. *Lipids in Health and Disease*.

[94] Wei Y, Wang D, Pagliassotti MJ (2007). Saturated fatty acid-mediated endoplasmic reticulum stress and apoptosis are augmented by trans-10, cis-12-conjugated linoleic acid in liver cells. *Molecular and Cellular Biochemistry*.

[95] Nakagawa T, Zhu H, Morishima N (2000). Caspase-12 mediates endoplasmic-reticulum-specific apoptosis and cytotoxicity by amyloid-*β*. *Nature*.

[96] Hetz C, Bernasconi P, Fisher J (2006). Proapoptotic BAX and BAK modulate the unfolded protein response by a direct interaction with IRE1alpha. *Science*.

[97] Upton J-P, Wang L, Han D (2012). IRE1*α* cleaves select microRNAs during ER stress to derepress translation of proapoptotic caspase-2. *Science*.

[98] Walter P, Ron D (2011). The unfolded protein response: from stress pathway to homeostatic regulation. *Science*.

[99] Xu C, Bailly-Maitre B, Reed JC (2005). Endoplasmic reticulum stress: cell life and death decisions. *Journal of Clinical Investigation*.

[100] Němcová-Fürstová V, James RF, Kovář J (2011). Inhibitory effect of unsaturated fatty acids on saturated fatty acid-induced apoptosis in human pancreatic ك-cells:activation of caspases and ER stress induction. *Cellular Physiology and Biochemistry*.

[101] Bäckhed F, Ding H, Wang T (2004). The gut microbiota as an environmental factor that regulates fat storage. *Proceedings of the National Academy of Sciences of the United States of America*.

[102] Tsukumo DM, Carvalho BM, Carvalho-Filho MA, Mário JAS (2009). Translational research into gut microbiota: new horizons in obesity treatment. *Arquivos Brasileiros de Endocrinologia e Metabologia*.

[103] Cani PD, Neyrinck AM, Fava F (2007). Selective increases of bifidobacteria in gut microflora improve high-fat-diet-induced diabetes in mice through a mechanism associated with endotoxaemia. *Diabetologia*.

[104] Delzenne NM, Cani PD (2011). Interaction between obesity and the gut microbiota: relevance in nutrition. *Annual Review of Nutrition*.

[105] de Wit N, Derrien M, Bosch-Vermeulen H (2012). Saturated fat stimulates obesity and hepatic steatosis and affects gut microbiota composition by an enhanced overflow of dietary fat to the distal intestine. *The American Journal of Physiology, Gastrointestinal and Liver Physiology*.

[106] Machado MV, Cortez-Pinto H (2012). Gut microbiota and nonalcoholic fatty liver disease. *Annals of Hepatology*.

[107] Lira FS, Rosa JC, Pimentel GD (2012). Both adiponectin and interleukin-10 inhibit LPS-induced activation of the NF-*κ*B pathway in 3T3-L1 adipocytes. *Cytokine*.

[108] Masquio DC, de Piano A, Sanches PL (2012). The effect of weight loss magnitude on pro/anti-inflammatory adipokines and carotid intima-media thickness in obese adolescents engaged in interdisciplinary weight-loss therapy. *Clinical Endocrinology*.

